# PRL stimulates mitotic errors by suppressing kinetochore-localized activation of AMPK during mitosis

**DOI:** 10.1247/csf.22034

**Published:** 2022-11-05

**Authors:** Kajung Ryu, Atsushi Yoshida, Yosuke Funato, Daisuke Yamazaki, Hiroaki Miki

**Affiliations:** 1 Department of Cellular Regulation, Research Institute for Microbial Diseases, Osaka University, Suita, Osaka 565-0871, Japan; 2 Center for Infectious Disease Education and Research (CiDER), Osaka University, Suita, Osaka 565-0871, Japan

**Keywords:** cancer, AMPK, PRL, kinetochore, mitotic errors

## Abstract

Phosphatase of regenerating liver (PRL) is frequently overexpressed in various malignant cancers and is known to be a driver of malignancy. Here, we demonstrated that PRL overexpression causes mitotic errors that accompany spindle misorientation and aneuploidy, which are intimately associated with cancer progression. Mechanistic analyses of this phenomenon revealed dysregulation of the energy sensor kinase, AMP-activated protein kinase (AMPK), in PRL-induced mitotic errors. Specifically, immunofluorescence analysis showed that levels of phosphorylated AMPK (P-AMPK), an activated form of AMPK, at the kinetochore were reduced by PRL expression. Moreover, artificial activation of AMPK using chemical activators, such as A769662 and AICAR, in PRL-expressing cells restored P-AMPK signals at the kinetochore and normalized spindle orientation. Collectively, these results indicate the crucial importance of the activation of kinetochore-localized AMPK in the normal progression of mitosis, which is specifically perturbed by PRL overexpression.

## Introduction

Phosphatase of regenerating liver (PRL) is a family of three related proteins, PRL1–PRL3, in mammals (Diamond *et al.*, 1994; Zeng *et al.*, 1998). The PRL family of proteins have a tyrosine phosphatase domain and are anchored to the plasma membrane by prenylation at its C-terminus (Cates *et al.*, 1996). Gene expression analysis of human colorectal cancers revealed *PRL3* overexpression in all metastatic cancers examined (Saha *et al.*, 2001). Later, many histological studies on clinical samples reported a link between PRL overexpression and malignancy in various types of cancers (Bessette *et al.*, 2008). In addition, cultured cancer cells stably expressing PRL1 or PRL3 form more tumors in mice than their parental control cells (Zeng *et al.*, 2003), and gene disruption of *Prl3* in mice suppresses colorectal tumor formation induced by azoxymethane and dextran sodium sulfate (Zimmerman *et al.*, 2013). Collectively, these analyses indicate a role of PRL in promoting tumorigenesis.

Recent reports have shown that the interaction between PRL and cyclin M (CNNM) family Mg^2+^ transporter proteins, which comprise CNNM1–CNNM4 in mammals, is required for PRL-induced cancer malignancy (Wang *et al.*, 2003; Funato *et al.*, 2014; Hardy *et al.*, 2015; Gulerez *et al.*, 2016; Kozlov *et al.*, 2020). When bound to PRL, the Mg^2+^ efflux activity of CNNM proteins is suppressed, resulting in an increase in intracellular Mg^2+^ levels and a concomitant increase in the levels of ATP, a most major binding partner for Mg^2+^ (Funato *et al.*, 2014). Similar to PRL overexpression, CNNM4 knockdown in cultured cancer cells promotes tumor formation in mice, and *Cnnm4* disruption in mice heterogeneously deficient in *Apc*, which are predisposed to have benign intestinal polyps, stimulates malignant progression of the polyps to invasive adenocarcinomas. Moreover, intracellular Mg^2+^ and ATP levels were also significantly increased in cultured cells following CNNM4 knockdown (Funato *et al.*, 2014).

Cellular ATP levels affect the activation status of the key energy sensor molecule AMP-activated protein kinase (AMPK), which is activated by a reduction in ATP levels (Hardie, 2007). AMPK is a heterotrimeric serine/threonine kinase that consists of one catalytic α subunit and two regulatory β- and γ-subunits (Stapleton *et al.*, 1996). The energy status-dependent activity is regulated by phosphorylation at Thr172 in the α subunit by the liver kinase B1 (LKB1) or the calcium/calmodulin-dependent protein kinase kinase (CAMKK) protein family (Woods *et al.*, 2003; Hawley *et al.*, 2005). In addition, phosphorylated AMPK (P-AMPK) proteins have been reported to localize to various mitotic structures such as spindle poles, midzones, and midbodies, and kinetochores during mitosis (Vazquez-Martin *et al.*, 2011; Lu *et al.*, 2021). Studies using AMPK-knockdown or knockout mammalian cultured cells and *Ampk*-null *Drosophila melanogaster* mutants have revealed a role of AMPK in mitosis, since they have various mitotic defects, such as mitotic delay, chromosome misalignment, spindle misorientation, and chromosome missegregation, such as lagging or polyploid chromosomes (Lu *et al.*, 2021; Thaiparambil *et al.*, 2012; Lee *et al.*, 2007; Stauffer *et al.*, 2019). Mitotic errors are often associated with aneuploidy, defined as the loss or gain of chromosomes, which is found in approximately 90% of tumors and has been proposed to drive cancer progression (Ben-David and Amon, 2020; Vasudevan *et al.*, 2021).

We recently reported that PRL-expressing cells can proliferate actively even under acidic culture conditions of pH 6.5 (Funato *et al.*, 2020). The mechanism underlying this phenomenon involves PRL3 expression which promotes H^+^ extrusion to the extracellular surface by stimulating lysosomal exocytosis, thereby alleviating the intracellular acidification. In contrast, when PRL3-expressing cells were placed at pH 7.5, which is close to the physiological condition of pH 7.4, they ceased to proliferate. Further, cells died at pH 8.0. Therefore, the effect of PRL3 expression is ambivalent: it stimulates cell proliferation under acidic conditions, but suppresses them under alkaline conditions. However, the mechanism underlying this alkaline vulnerability of PRL-expressing cells remains unknown.

In this study, we explored the death mechanism of PRL3-expressing cells at pH 8.0, and found that cell death specifically occurred during mitosis with the characteristic mitotic errors, such as spindle misorientation. Similar mitotic defects were also observed at pH 7.5, close to physiological conditions, and continuous culture of PRL3-expressing cells at pH 7.5 for consecutive 7 days significantly augmented aneuploidy levels. Furthermore, we found that P-AMPK, an activated form of AMPK, was localized not only in previously reported mitotic structures but also at kinetochores during metaphase, which was specifically perturbed by PRL expression. Collectively, PRL overexpression can drive mitotic errors by suppressing P-AMPK activity at kinetochores and impeding chromosome assembly during metaphase.

## Materials and Methods

### Cell culture, transfection, and synchronization

HeLa, MDCK, and MDCK-derived cell lines that express GFP-PRL3 in a doxycycline (Dox)-inducible manner, generated in our previous study (Kojima *et al.*, 2019), were maintained in Dulbecco’s modiﬁed Eagle’s medium (DMEM, Nissui 05919) supplemented with 10% fetal bovine serum (FBS) and antibiotics at 37°C under 5% CO_2_ and 95% air conditions. MDCK-derived cell lines expressing Myc-PRL3 in a Dox-inducible manner were established using the Tet-On system plasmids pcDNA6/TR and pcDNA4/TO (Invitrogen), as described previously (Kojima *et al.*, 2019). Cell culture under pH-fixed conditions was performed at 37°C without CO_2_ supply, using pH-fixed medium prepared by adding 25 mM HEPES (pH was adjusted with HCl or NaOH). Cells were seeded and cultured for 24 h under the standard growing condition, and then, they were subjected to pH-fixed condition and cultured with or without 2 μg/ml of Dox for 14 h. To synchronize cells at mitosis for experiments shown in Supplementary Fig. 3, cells were incubated with 500 nM nocodazole for 16 h.

### Mice and isolation and culture of colonic epithelial cells

*Cnnm4* knockout mice were generated in our previous study (Yamazaki *et al.*, 2019). Male and female mice between 4 and 8-months-old were used in the experiments. Mice were treated in accordance with the animal experimental guidelines issued by the Science Council of Japan. This study was approved by the Institutional Review Board of Osaka University. Murine colonic crypts were isolated and cultured as described in the previous study (Yamazaki *et al.*, 2019).

### Antibodies and chemicals

The anti-PRL rabbit polyclonal antibody was generated as previously described (Funato *et al.*, 2014). The anti-CNNM4 rabbit polyclonal antibody was generated as previously described (Yamazaki *et al.*, 2013). The following commercially available primary antibodies were used: α-tubulin mouse monoclonal (for staining culture cells, clone B-7, sc-5286), α-tubulin rat monoclonal (for staining organoids, clone YL1/2, sc-53029), Hec1 mouse monoclonal (clone C-11, sc-515550), Mad2 mouse monoclonal (for immunofluorescence staining analysis, clone 107-276-3, sc-65492), Mad2 mouse monoclonal (for immunoblotting analysis, clone 17D10, sc-47747), and c-Myc rabbit polyclonal (clone A14, sc-789) antibodies from Santa Cruz Biotechnology; γ-tubulin rabbit polyclonal (T5192) antibodies from Sigma-Aldrich; CREST human auto-immune (HCT-0100) antibodies from ImmunoVision; AMPKα rabbit polyclonal (#2532S), phospho-AMPKα T172 rabbit monoclonal (clone 40H9, #2535S), and cyclin B1 mouse monoclonal (clone V152, #4135S) antibodies from Cell Signaling Technology; BubR1 sheep polyclonal (ab28193) antibodies from Abcam; β-actin mouse monoclonal (clone 2D4H5, 66009-1-Ig) antibodies from Proteintech. The following commercially available secondary antibodies were used: Alexa 488-conjugated anti-mouse IgG (A11029), anti-rabbit IgG (A11034), anti-rat IgG (A11006), and anti-sheep IgG (A11015) antibodies, Alexa 568-conjugated anti-rabbit IgG (A11036) and anti-human IgG (A21090) antibodies, and Alexa 633-conjugated anti-mouse IgG (A21052) antibodies from Invitrogen for immunofluorescence staining. Horseradish peroxidase-conjugated anti-sheep IgG (H+L) (31480) from Pierce; alkaline phosphatase-conjugated anti-mouse IgG (S3721) and anti-rabbit IgG (S3731) from Promega for immunoblotting analysis. We used the following commercially available chemicals: Dox (14203-91) and Hoechst 33342 (0491581) from Nacalai Tesque, KaryoMAX COLCEMID solution (15212-012) from Life Technologies, AICAR (015-22531) from Wako, A769662 (A-1803) from LC laboratories, compound C (171260) from Merck, and nocodazole (M-1404) from Sigma Aldrich.

### Time-lapse imaging of culture cells

Culture cells were stained with 2 μM Hoechst 33342 diluted in pH-fixed media (pH 8.0) for 15 min at 37°C to visualize DNA. After washing three times with PBS, the medium was replaced with fresh medium, and fluorescent and phase-contrast images were obtained every 20 min for 24 h using an Olympus IX81 microscope.

### Immunostaining of culture cells

To stain for γ-tubulin and α-tubulin, cells cultured on glass coverslips were fixed in ice-cold methanol for 15 min at –20°C. After washing three times with PBS, the cells were blocked in PBS containing 5% normal goat serum and 0.3% TritonX-100 (TX-100) for 1 h. The cells were then incubated overnight at 4°C with γ-tubulin (1:1000) and α-tubulin (1:100) antibodies diluted in PBS containing 1% bovine serum albumin (BSA) and 0.3% TX-100. To stain for Myc or P-AMPK, cells were fixed with PBS containing 4% paraformaldehyde (PFA) for 10 min at room temperature (RT), washed three times with PBS, and permeabilized with 0.2% TX-100 in PBS for 5 min. After washing three times with PBS, the cells were blocked in PBS containing 10% BSA and 3% FBS for 1 h, and incubated overnight with c-Myc antibodies (1:100) or P-AMPK antibodies (1:100) diluted in PBS containing 10% BSA and 3% FBS at 4°C. To stain for Hec1, cells were fixed with PBS containing 2% PFA for 10 min at RT, washed three times with PBS, and permeabilized with 0.2% TX-100 in PBS for 5 min at RT. The cells were blocked in PBS containing 2% BSA and 0.5% TX-100 for 30 min at RT, washed three times with PBS, and incubated overnight at 4°C with Hec1 antibodies (1:100) diluted in PBS containing 2% BSA and 0.5% TX-100. To stain for BubR1, cells were fixed with PBS containing 1% PFA for 10 min at RT, washed three times with PBS, and permeabilized with 0.2% TX-100 in PBS for 5 min at RT. After washing three times with PBS, the cells were blocked in PBS containing 1% BSA for 30 min at 37°C, and incubated overnight at 4°C with BubR1 antibodies (1:200) diluted in PBS containing 1% BSA. To stain for Mad2, cells were fixed with 1% formaldehyde diluted in PBS, washed three times with PBS, permeabilized with 0.2% TX-100 in PBS for 5 min at RT, and then washed three times with PBS. The cells were blocked in PBS containing 3% BSA for 30 min at RT and incubated overnight at 4°C with Mad2 antibodies (1:50) diluted in 3% BSA in PBS. For visualizing kinetochores, CREST antibodies (1:1000) were also included in the incubation step with each of the above primary antibodies. After incubation with primary antibodies, the cells were washed three times with PBS and incubated with fluorophore-conjugated secondary antibodies (1:2000), 4,6-diamidino-2-phenylindole (DAPI, Roche, 10236276001, 1:1000) to stain for DNA, and rhodamine-conjugated phalloidin (Wako 165-21641, 1:250) to stain for filamentous actin (F-actin) for 1 h at RT. After washing three times with PBS, glass coverslips were mounted on glass slides and observed using a confocal scanning laser microscope (FLUOVIEW FV1000, Olympus).

### Staining of mouse colon tissues

Colon tissues were dissected after the mice were sacrificed by cervical dislocation and fixed with 4% PFA in PBS overnight at RT. Tissues were then soaked in PBS containing 30% sucrose for 12 h at RT, embedded in OCT compound (Sakura Finetechnical), and frozen in liquid nitrogen. Frozen samples were sliced using a cryostat (Leica), mounted on glass slides, and air-dried. For spindle orientation analysis, 20 μm thick tissue section slides were prepared and permeabilized with 0.2% TX-100 in PBS for 5 min. After washing three times with PBS, tissues were blocked in PBS containing 3% FBS and 10% BSA for 1 h at RT, and incubated with DAPI (1:1000) and rhodamine-conjugated phalloidin (1:250) for 1 h at RT. Glass coverslips were mounted on tissue section slides and observed using a confocal scanning laser microscope (FLUOVIEW FV1000, Olympus). For chromosome segregation analyses, 5 μm thick tissue sections on slides were fixed with 3.7% formaldehyde in PBS for 10 min at RT, and stained with hematoxylin (Mayer’s hematoxylin (Wako)) and eosin (0.1% eosin Y (Wako)) (H&E). Glass coverslips were mounted on the slides and observed under a microscope (BX41 equipped with a DP73 camera, Olympus).

### Immunostaining of colonic organoids

Colonic organoids embedded in Matrigel (Corning 356231) were released using Cell Recovery Solution (Corning 354253) and fixed with 1 ml of formaldehyde/methanol (2.96% of formaldehyde in methanol) for 15 min at –20°C. After fixation, organoids were permeabilized with 1% TX-100 for 3 min at RT. After blocking with PBS containing 3% FBS, 10% BSA, and 0.5% TX-100 for 1 h at RT, the organoids were incubated overnight at 4°C with α-tubulin antibody (1:25) and γ-tubulin antibody (1:500) diluted in PBS containing 0.5% TX-100. After washing, the organoids were incubated with secondary antibodies (1:2000) and DAPI (1:1000) diluted in PBS containing 3% FBS, 10% BSA, and 0.5% TX-100 for 1 h at RT. A confocal scanning laser microscope (FLUOVIEW FV1000, Olympus) was used for observation.

### Analyses of spindle orientation and spindle pole distance

To analyze the spindle orientation and spindle pole distance of cultured cells, cells were stained with DAPI and γ-tubulin or P-AMPK antibodies. When needed, the cells were incubated with 5 mM AICAR, 300 μM A769662 for 30 min, or 20 μM compound C for 10 min or 20 min before fixation. Horizontal section images (X-Y images) of mitotic cells at metaphase were obtained from the bottom to the top of the cells with the interval of 0.5 μm using a confocal scanning laser microscope (FLUOVIEW FV1000, Olympus). The X-Y images were then stacked, and vertical section images (X-Z images), which contained two spindle poles, were constructed using the software FV10-ASW (Olympus). The horizontal and vertical distances between two spindle poles (xy, and z, respectively) were measured using ImageJ software (National Institutes of Health). The spindle angle (α) was then calculated using the inverse trigonometric function (arctan).

$$\alpha =\tan^{-1}\;(\frac{z}{xy})$$

The distance between the two spindle poles (*d*) was calculated as *d*^2^ = *z*^2^ + *xy*^2^.

To analyze the spindle orientation of cells in mouse colon tissues, 20 μm thick tissue section slides were stained with DAPI and rhodamine-conjugated phalloidin. Mitotic cells at metaphase in the crypts were observed using a confocal scanning laser microscope (FLUOVIEW FV1000, Olympus). The angle between the orthogonal vector of the metaphase plate and the line parallel to the basement membrane was measured using ImageJ. To analyze the spindle orientation of cells in colonic organoids, these were stained with α-tubulin, γ-tubulin antibodies, and DAPI. The centroid and circularity of each organoid were determined by tracing the basal surface using the selection tool of ImageJ. Organoids with circularity over 0.9 were used for analysis. The angle between the line connecting the two spindle poles (spindle axis) and the line connecting the centroid and midpoint of the spindle axis was measured using ImageJ.

### Chromosome segregation analyses

To analyze chromosome segregation in cultured cells, cells were stained with α-tubulin, CREST antibodies, and DAPI, and then mitotic cells at anaphase were observed using a confocal scanning laser microscope (FLUOVIEW FV1000, Olympus). Anaphase cells with chromosomes left in the spindle midzone were defined as missegregated cells. Chromosomes left at the spindle midzone that were positive for CREST were counted as lagging chromosomes, and chromosomes negative for CREST were counted as acentric chromatids. To analyze chromosome segregation of cells in the colon tissues, 5 μm thick tissue section slides were stained using the standard H&E method. The anaphase cells in the crypt were observed under a microscope (BX41 equipped with a DP73 camera, Olympus) and cells with chromosomes left at the spindle midzone were counted as missegregated cells.

### Karyotyping

Cells were cultured in pH-fixed media for 7 days in the presence or absence of 2 μg/ml of Dox, with medium replacement every 2 days. The cells were incubated with 0.05 μg/ml KaryoMAX COLCEMID solution for 1 h to accumulate mitotic cells and harvested after dissociating cells with trypsin for 5 min at 37°C. The cells were suspended in a hypotonic solution (75 mM KCl) for 20 min at RT and incubated with a fixative solution (3:1 methanol:acetic acid) for 3 min at RT. The suspension was centrifuged at 12,000 rpm for 3 min, and the pellet was washed twice with a hypertonic solution. Finally, the pellet was suspended in 1 ml of hypertonic solution. The mixture was then dropped onto glass slides and air-dried for a few minutes. The cover glasses were mounted on glass slides with a mount solution (0.5% *p*-phenylenediamine in 20 mM Tris-HCl (pH 8.8), 90% glycerol) containing DAPI. Colonic organoids were incubated with 0.05 μg/ml of KaryoMAX COLCEMID solution for 4 h at 37°C and released from Matrigel using Cell Recovery Solution. The organoids were treated with TrypLE (Gibco 12604013) for 3 min and the dissociated cells were treated as described above. A confocal scanning laser microscope (FLUOVIEW FV1000, Olympus) was used for observation.

### Cell cycle analysis by Fluorescence-Activated Cell Sorter (FACS)

Cells were trypsinized after 14 h or 24 h of Dox treatment, and then fixed with ice-cold 70% ethanol. Cells were then pelleted and stained with DAPI/0.1% Triton-X in PBS for 30 min at room temperature. FACS analysis was performed by Attune NxT flow cytometer (Invitrogen) in three independent experiments and percentage of cell population in different phases of the cell cycle was analyzed using Attune NxT software.

### Quantification of Hec1 and BubR1

To quantify the fluorescence intensity of Hec1 or BubR1 in kinetochores, the cells were stained with DAPI, CREST, and Hec1 or BubR1 antibodies. Z-stacked images (0.2 μm/section) of mitotic cells at prometaphase were converted to maximum intensity projection (z projection) using ImageJ. Regions of the kinetochore were selected based on the CREST signal. The signal intensity of Hec1 or BubR1 at the kinetochore was measured and divided by that of CREST.

### Statistics

Data are presented as mean or mean ± SEM, and *p* values were calculated using Prism 6 software (GraphPad) or R software. One-way ANOVA with Tukey’s multiple comparison and two-tailed unpaired *t*-test were performed with Prism 6 software, and Chi-square test and Fisher’s exact test with Bonferroni correction for multiple comparison was performed with R software, as described in the figure legends.

## Results

### PRL3 expression causes mitotic errors

**Fig. 1:**
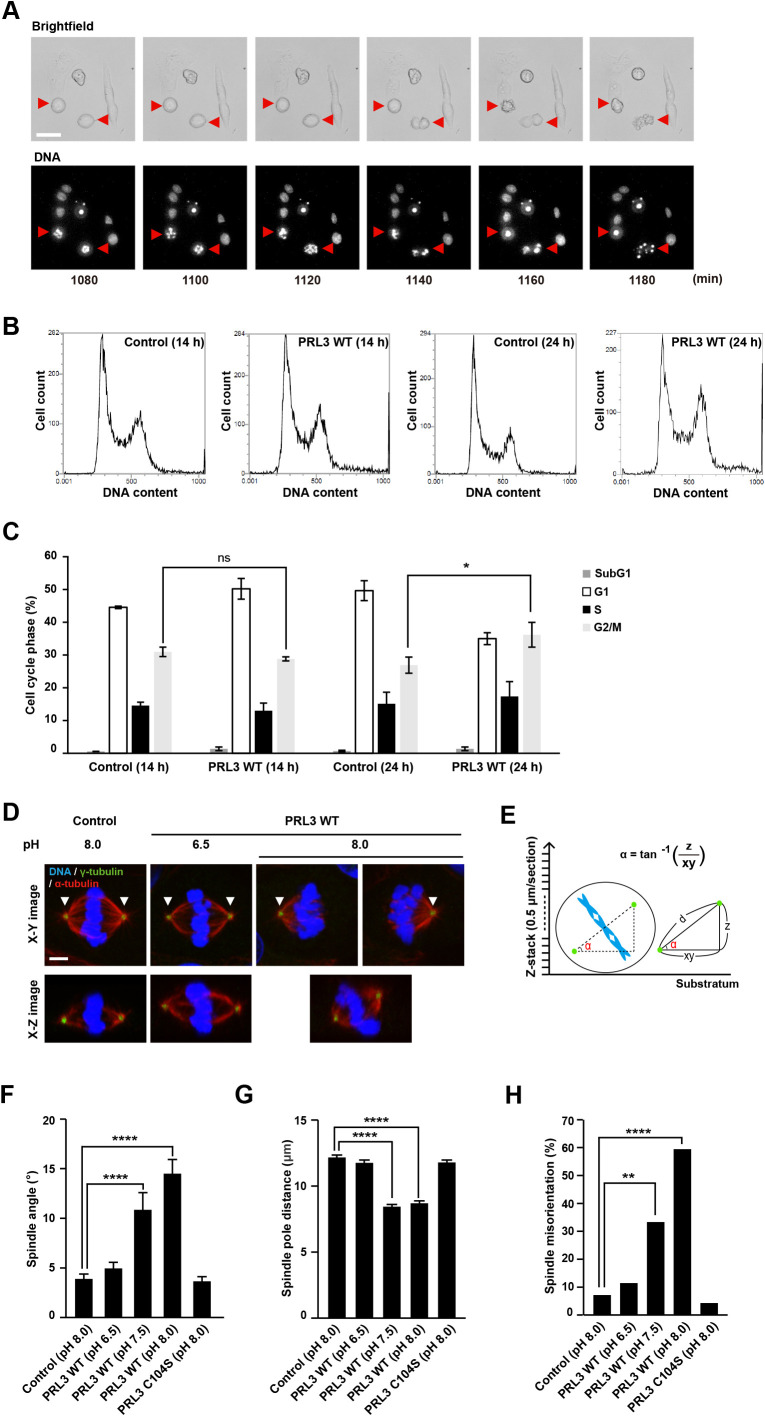
Spindle orientation is perturbed in PRL3-expressing cells (A) MDCK-derived cells that express PRL3 in a doxycycline (Dox)-inducible manner were stained with Hoechst 33343 diluted in pH-fixed media (pH 8.0) in the presence of Dox. Phase-contrast (upper) and fluorescent (Hoechst 33343, lower) images were obtained every 20 min. Red arrowheads indicate dying cells during mitosis. Scale bar: 50 μm. Numbers in the bottom of each frame indicate the time after treatment with Dox under the fixed pH condition (minutes). (B) Representative histograms of DNA content of control and Dox-inducible PRL3-expressing cells cultured under pH-fixed media (pH 8.0) for 14 or 24 h in the presence of Dox. The cells were fixed and stained with DAPI before determining DNA content using FACS. (C) Quantification of the portion of cells in SubG1, G1, S, G2/M phase, respectively. Data are shown as mean ± SEM, n = 3. The *p* values were calculated via two-way ANOVA with Tukey’s multiple comparison. ns: non-significant, *p<0.05 against control cells. (D) Control MDCK cells or Dox-inducible PRL3-expressing MDCK cells were cultured under indicated pH conditions 14 h in the presence of Dox. The cells were fixed and stained with DAPI (blue), anti-γ-tubulin (green), and anti-α-tubulin (red) antibodies. Upper panels show horizontal section images (X-Y images) of the mitotic cells at metaphase and lower panels show vertical section images (X-Z images) which contain two spindle poles. White arrowheads indicate spindle poles. Scale bar: 5 μm. (E) Scheme depicting spindle angle (α) and spindle pole distance (d) measurement in fixed cells: vertical distance (z) and horizontal distance (xy) between two spindle poles. (F–G) Quantification of the spindle angle (F) and spindle pole distance (G) of mitotic cells at metaphase. Data are shown as mean ± SEM, n = 60–70 cells. The *p* values were calculated via one-way ANOVA with Tukey’s multiple comparison. ****p<0.0001 against control cells. (H) Percentage of spindle misoriented cells having spindle angle of more than 10° were counted. The *p* values were calculated via Fisher’s exact test with Bonferroni correction for multiple comparison. **p<0.01, ****p<0.0001 against control cells.

**Fig. 2:**
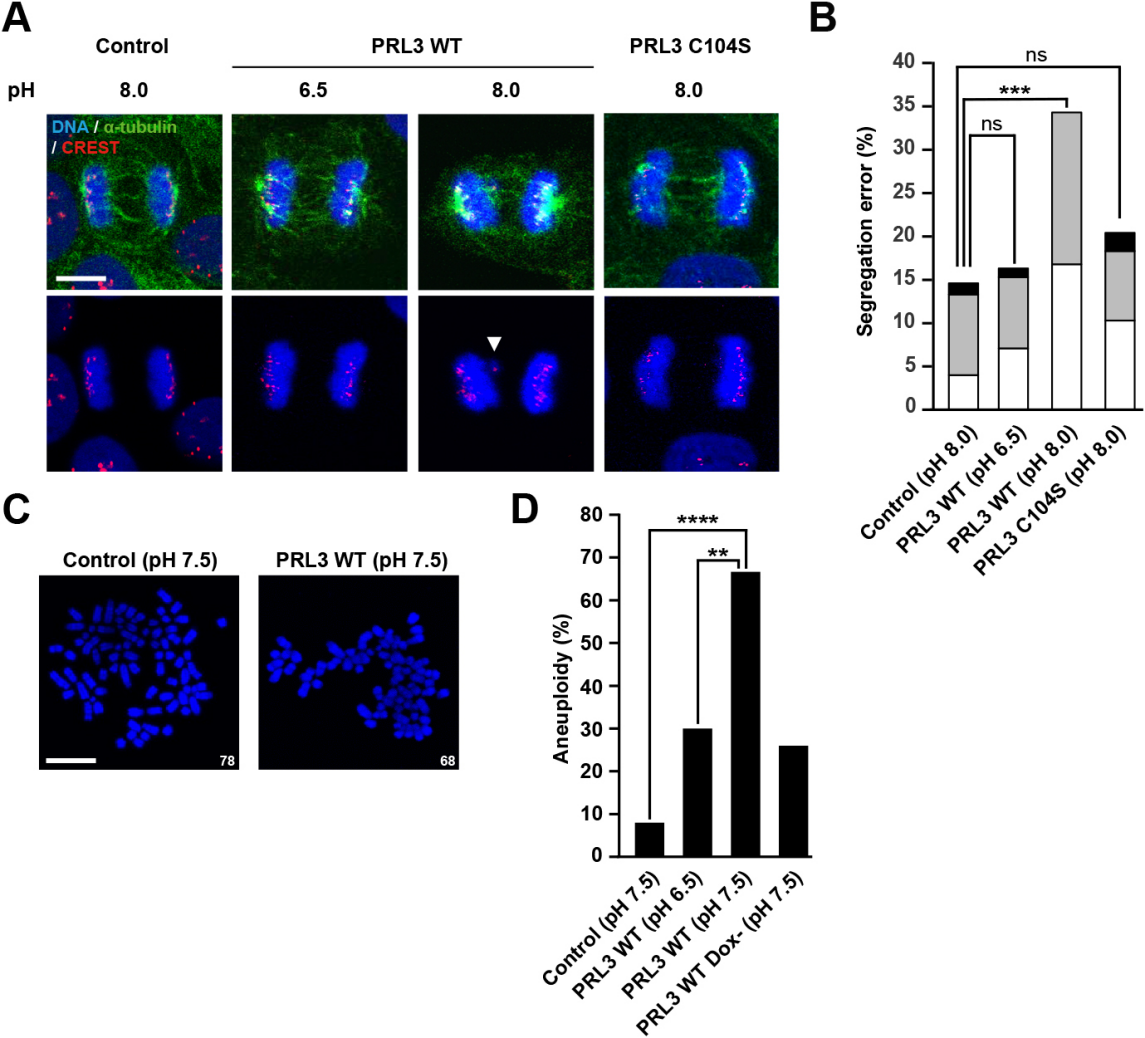
Chromosome segregation errors in PRL3-expressing cells (A) Control MDCK cells or Dox-inducible PRL3-expressing MDCK cells were cultured under indicated pH conditions for 14 h in the presence of Dox. Cells were stained with DAPI (blue), anti-α-tubulin (green), and anti-CREST (red) antibodies, and mitotic cells at anaphase were observed. The fluorescence signal of α-tubulin was excluded to visualize chromosomes (lower panel). White arrowhead indicates lagging chromosome. Scale bar: 5 μm. (B) Quantification of percentage of anaphase cells with lagging chromosomes (white bar), acentric chromatids (grey bar), and bridges (black bar). The *p* value was calculated via Fisher’s exact test with Bonferroni correction for multiple comparison, n = 97–151 cells. ***p<0.001, ns: non-significant against control cells. (C) The indicated cells were cultured under indicated pH conditions for 7 days, with media replaced every 2 days. Mitotic chromosome spreads from indicated cell lines were prepared. Chromosomes were stained with DAPI (blue) and the number of chromosomes in each cell was counted. Scale bar: 10 μm. Number in the lower right corner indicates number of chromosomes. (D) Quantification of percentage of aneuploid cells. Cells with a chromosome number that deviated from the modal chromosome number (2n = 78) by ≥10% were defined as aneuploid cells. The *p* value was calculated via Fisher’s exact test with Bonferroni correction for multiple comparison, n = 50–51 cells. **p<0.01, ****p<0.0001.

We have previously reported that PRL3-expressing cells alter their response to environmental pH; cells cease to proliferate at a standard pH of 7.5, proliferate more rapidly at pH 6.5, and die under alkaline conditions of pH 8.0 (Funato *et al.*, 2020). To investigate how PRL3-expressing cells died at pH 8.0, they were observed using time-lapse microscopy. The cells were stained with Hoechst 33343 to visualize DNA and then cultured in pH 8.0-fixed media containing doxycycline (Dox) to induce the expression of GFP-PRL3. The images were taken for 24 h with 20 min interval (Supplementary Video 1 and 2). We noticed that PRL3-expressing cells did not divide properly (Fig. 1A). More specifically, mitotic cells died successively after 13 h of Dox induction (Supplementary Video 2). To investigate in more detail, we traced 20 mitotic cells, which have spherical morphology and have condensed DNA, after 13 h of Dox treatment. 14 cells in PRL3-expressing cells were found to die with nucleus fragmentation (mitotic catastrophe, Supplementary Table 1), while all the control cells divided normally. In addition, duration of mitosis in the remaining mitotic PRL3-expressing cells (90 min) was longer than control cells (56 min). Furthermore, cell cycle analysis using FACS showed the percentages of G2/M phase portion in PRL3-expressing cells were increased compared to control cells after 24 h of Dox induction (Fig. 1B–C). These results led us to examine the formation of the mitotic spindle, which is an apparatus that aligns chromosomes, at a relatively early time point (14 h after Dox treatment). MDCK cells that can inducibly express Myc-tagged PRL3 proteins with Dox treatment were generated for performing multiple staining (Supplementary Fig. 1), and were stained for α-tubulin and γ-tubulin to observe spindle formation using a confocal microscope. Normally, two spindle poles, which can be visualized by γ-tubulin staining, are observed in the same horizontal section image (X-Y image) at the metaphase of MDCK cells (Reinsch and Karsenti, 1994). In contrast, the spindle poles in PRL3-expressing cells were observed separately in different confocal section images (Fig. 1D, upper). Vertical section images (X-Z images) containing two spindle poles showed that PRL3-expressing cells had misoriented spindles with their axes tilted relative to the substratum plane (Fig. 1D, lower). In contrast, cells had normal spindle orientation when cultured in pH 6.5 in which they can proliferate most actively, as reported previously (Funato *et al.*, 2020). To quantify spindle misorientation, we measured the spindle angle (α) and spindle pole distance (d), as shown in Fig. 1E. The results showed that PRL3-expressing cells at pH 8.0 had increased spindle angle (Fig. 1F) and reduced spindle pole distance (Fig. 1G) compared to control cells. We also counted the number of spindle misoriented cells, which were defined those having spindle angle of more than 10 degrees, and confirmed that the rate of those cells was increased in PRL3-expressing cells than control cells (Fig. 1H). We also performed similar quantitative experiments by culturing PRL3-expressing cells in pH 7.5, which is close to the physiological condition inside mammalian bodies (Anemone *et al.*, 2019), and found that they also showed misoriented spindles with an increased spindle angle and reduced spindle pole distance. Therefore, the mitotic defect caused by PRL expression is considered to occur not only under alkaline conditions, but also under physiological pH conditions. Moreover, we tested the effects of PRL3-C104S, a mutant that has been shown to be biologically inactive to induce cancer malignancy (Funato *et al.*, 2014; Guo *et al.*, 2004). The results showed that the C104S mutant was ineffective in inducing spindle misorientation, suggesting the importance of spindle misorientation in PRL3-induced cancer malignancy. Next, we examined the fidelity of chromosome segregation, as spindle misorientation is associated with chromosome missegregation (Nam *et al.*, 2015). The cells were stained for chromosomes, α-tubulin, and CREST, which is known to localize at kinetochores (Brenner *et al.*, 1981), and were observed at anaphase during mitosis (Fig. 2A). We noticed that PRL3-expressing cells cultured at pH 8.0 tended to have some chromosomes left at the spindle midzone (indicated with an arrowhead), whereas control cells and PRL3-expressing cells cultured at pH 6.5 mostly segregated their chromosomes properly. To confirm these findings, we quantified chromosome missegregation by counting the number of anaphase cells with midzone-left chromosomes: CREST-positive lagging chromosomes or CREST-negative acentric chromatids (Cimini *et al.*, 2001), or bridges, string-like chromatin threads that connect two masses of chromosomes (Gisselsson, 2008). As expected, PRL3-expressing cells at pH 8.0 showed higher levels of chromosome missegregation, having bridges, lagging chromosomes and acentric chromatids, than control cells (Fig. 2B). In contrast, no significant augmentation in chromosome missegregation was observed in PRL3-expressing cells cultured at pH 6.5 or PRL3-C104S-expressing cells. Because chromosome missegregation can result in the gain or loss of chromosomes, a state called aneuploidy, we counted the number of chromosomes in the cells. For this purpose, we cultured PRL3-expressing cells for 7 consecutive days at pH 7.5 (because they died at pH 8.0). Thereafter, the cells were collected and their chromosomes were spread and stained with DAPI (Fig. 2C). The results showed that PRL3-expressing cells had eight-fold higher levels of aneuploidy than control cells (Fig. 2D: Cells with a number of chromosomes that deviated over 10% from the modal chromosome numbers (2n = 78) were defined as aneuploid). When PRL3-expressing cells were cultured at pH 6.5, the aneuploidy level significantly decreased, indicating the pH dependency of this phenomenon. Collectively, these results imply that PRL3 overexpression can induce aneuploidy, which is a hallmark of cancer (Hanahan and Weinberg, 2011), under physiological pH conditions.

### Cnnm4 knockout causes mitotic errors

**Fig. 3:**
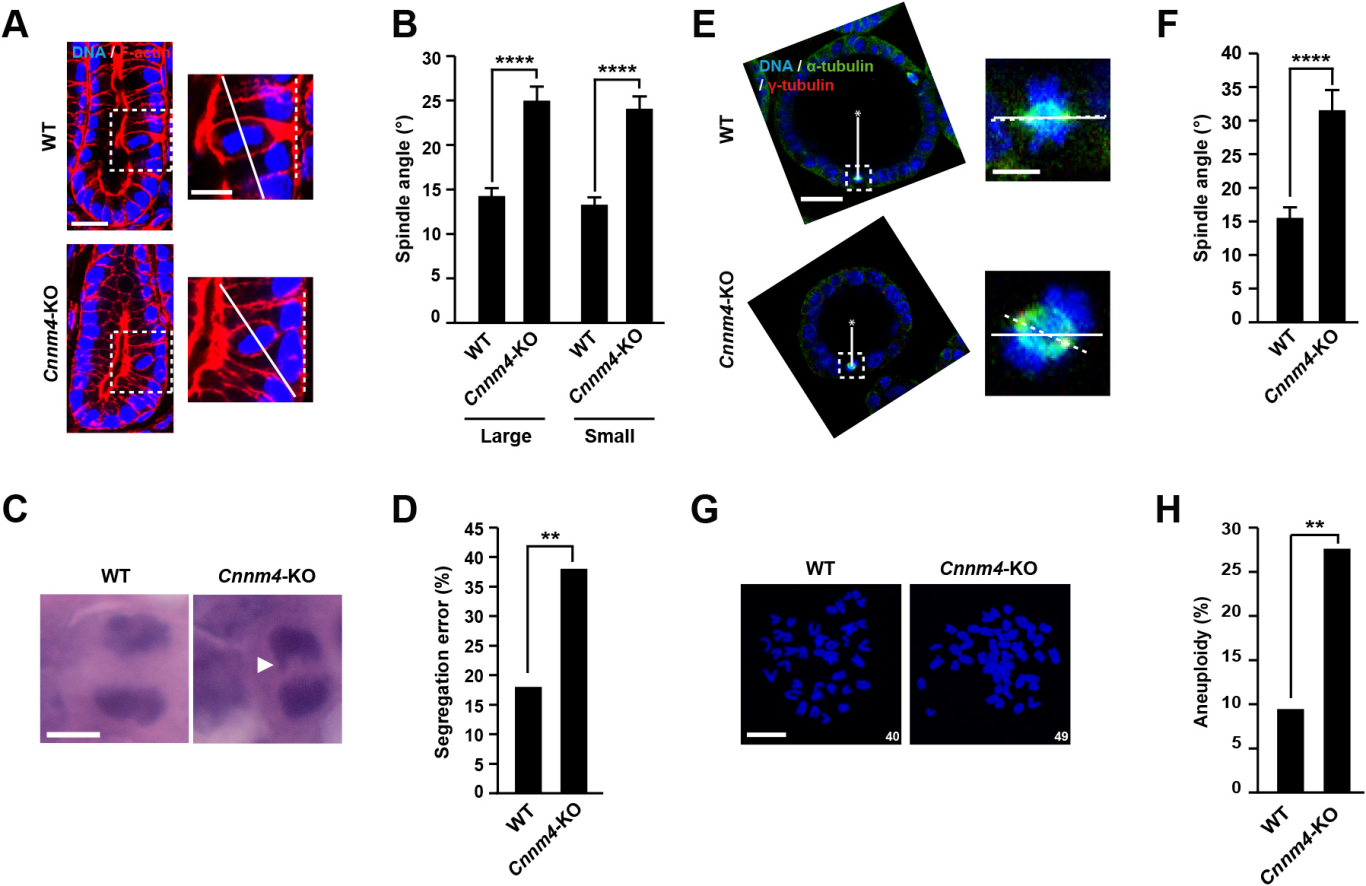
Mitotic errors in *Cnnm4*-KO colon epithelia (A) Colon tissue sections of wild-type (WT) and *Cnnm4*-KO mice were stained with DAPI (blue) and rhodamine-conjugated phalloidin (F-actin, red). Mitotic cells at metaphase in the crypts were observed (right panel: enlarged view of boxed area). Solid and dashed lines indicate the spindle axis or basement membrane of the crypts, respectively. Scale bar: 20 μm (left, original image) or 10 μm (right, enlarged image). (B) Quantification of the spindle angle. The spindle angle between the two lines (dashed and solid lines) indicated in (A) was measured. Data are shown as mean ± SEM, n = 106–132 cells (large intestine) or n = 124–133 cells (small intestine). The *p* values were calculated via two-tailed unpaired *t*-test. ****p<0.0001 against WT. (C) Colon tissue sections were subjected to H&E staining and mitotic cells at anaphase were observed. Arrowhead indicates missegregated chromosome. Scale bar: 5 μm. (D) Quantification of the chromosome segregation error. The *p* values were calculated via Chi-square test, n = 84–95 cells (3 mice). **p<0.01 against WT. (E) Colonic organoids derived from indicated mouse strains were stained with DAPI (blue), anti-γ-tubulin (green), and anti-α-tubulin (red) antibodies. Asterisk indicates the centroid of the respective organoid and solid lines connect the centroid and the center of the spindle. Right panel shows enlarged view of the boxed area and dashed line indicates spindle axis. Vertical solid line indicates a line that connects the centroid of organoid to the center of the spindle. Horizontal solid line is perpendicular to vertical solid line. Scale bar: 30 μm (left, original image) or 5 μm (right, enlarged image). (F) Quantification of the spindle angle of metaphase cells in the colonic organoids from indicated mouse strains. The spindle angle between the horizontal solid line and dashed line, which are indicated in (E), was measured and absolute value of the angle after subtracting 90° was used. Data are shown as mean ± SEM, n = 60 cells. The *p* values were calculated via two-tailed unpaired *t*-test. ****p<0.0001 against WT. (G) Mitotic chromosome spread from indicated colonic organoids were prepared. Chromosomes were stained with DAPI (blue) and the number of chromosomes in each cell was counted. Scale bar: 10 μm. Number in the lower right corner indicates number of chromosomes. (H) Quantification of percentage of aneuploid cells for colonic organoids from indicated mouse strains. Cells with a chromosome number that deviated from the modal chromosome number (2n = 40) by ≥10% were defined as aneuploid cells. The *p* value was calculated via Chi-square test, n = 95–105 cells. **p<0.01 against WT.

PRL directly binds to the CNNM Mg^2+^ transporter and inhibits Mg^2+^ efflux, which drives malignant progression of cancers (Funato *et al.*, 2014). As CNNM4 is strongly expressed in the intestinal epithelia, especially in the colon, we investigated mitotic errors *in vivo* using intestinal tissues obtained from wild-type (WT) and *Cnnm4*-knockout (*Cnnm4*-KO) mice (Yamazaki *et al.*, 2019). The loss of CNNM4 proteins in the *Cnnm4*-KO mouse sample was confirmed by immunoblotting analyses (Supplementary Fig. 2). Fixed tissue sections were stained with DAPI and rhodamine-conjugated phalloidin, and mitotic cells at metaphase in the crypts were observed (Fig. 3A). The spindle angle between the spindle axis (solid line) and the basement line (dashed line) in enlarged images was significantly larger in both the large and small intestines obtained from *Cnnm4*-KO mice than in those from WT mice (Fig. 3A–B). Next, we examined the fidelity of chromosome segregation. Fixed tissue sections were stained with H&E and mitotic cells were observed at anaphase (Fig. 3C). The cells from *Cnnm4*-KO mice had higher levels of chromosome missegregation than those from WT mice (Fig. 3D). Colonic epithelia can be cultured *in vitro* by forming organoids that maintain polarity with proliferating and differentiated populations of epithelial cells (Sato *et al.*, 2011). Therefore, crypts were isolated from the colon epithelia of WT mice and *Cnnm4*-KO mice and embedded in three-dimensional matrices for organoid culture. The loss of CNNM4 proteins in *Cnnm4*-KO mouse-derived organoids was confirmed by immunoblotting analyses (Supplementary Fig. 2). To investigate spindle orientation, organoids were stained for α-tubulin and γ-tubulin, and mitotic cells were observed during metaphase (Fig. 3E). The angles between the spindle axis (dashed line) and the basement line (horizontal solid line) in the enlarged images were measured. As expected, cells in organoid cultures derived from *Cnnm4*-KO mice had larger spindle angles than those from WT mice (Fig. 3F). As cells in organoid culture can be arrested during mitosis, we investigated the levels of aneuploidy (Fig. 3G). The results showed that cells in organoids from *Cnnm4*-KO mice had higher levels of aneuploidy than those from WT mice (Fig. 3H: Cells with a number of chromosomes that deviated over 10% from the modal chromosome numbers (2n = 40) were defined as aneuploid). Overall, these results indicate that *Cnnm4*-KO results in similar mitotic errors, consistent with the role of PRL in suppressing the function of CNNM proteins.

### Activation of AMPK at kinetochores in mitotic fidelity

**Fig. 4:**
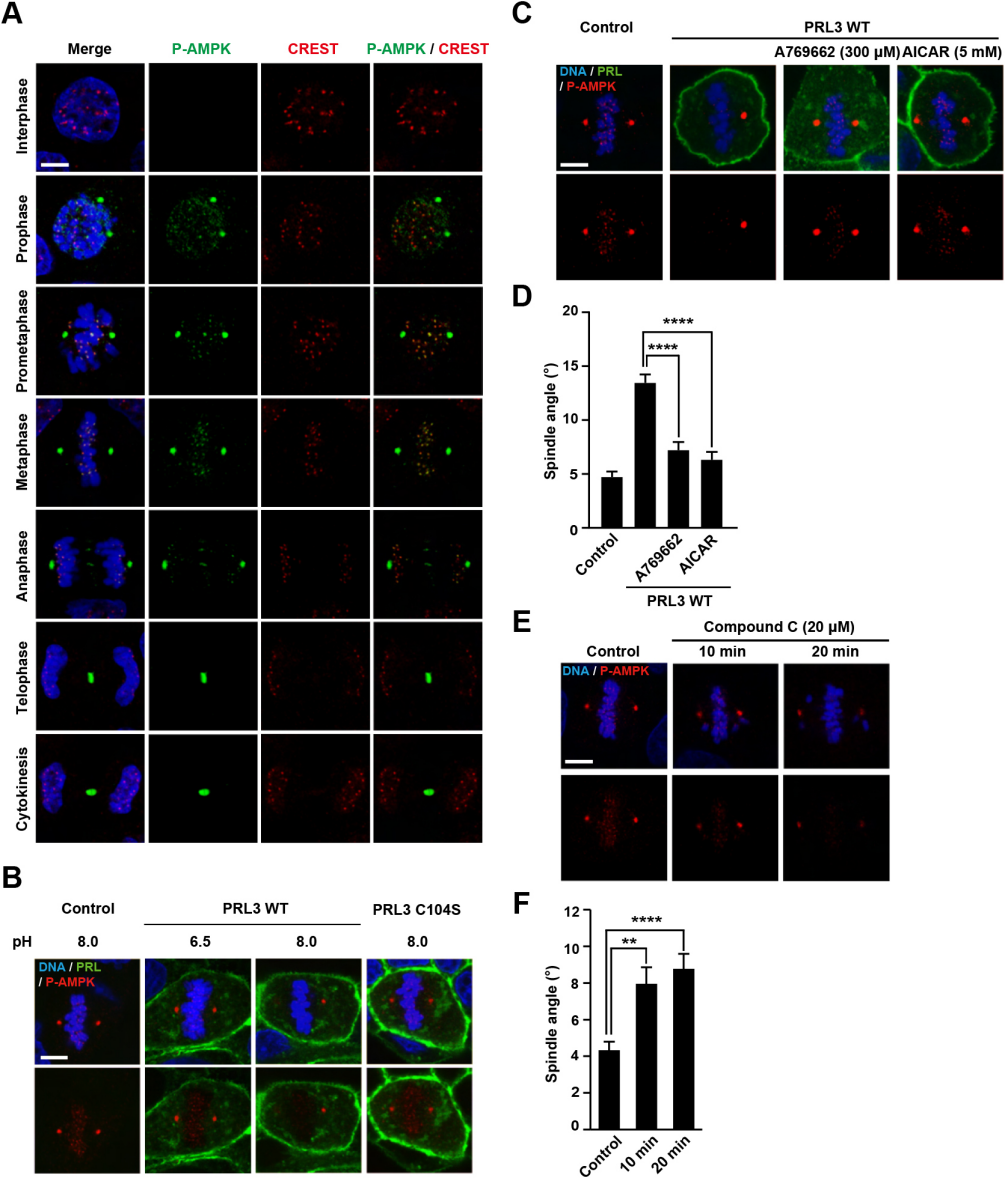
Importance of P-AMPK at kinetochores in mitotic fidelity (A) MDCK cells were cultured under pH-fixed condition (pH 7.5) for 14 h. The cells were stained with DAPI (blue), anti-P-AMPK (green), and anti-CREST (red) antibodies. Cells at indicated phases were observed. Scale bar: 5 μm. (B) Control MDCK cells or Dox-inducible PRL3-expressing MDCK cells cultured under pH-fixed condition (pH 6.5 and 7.5) for 14 h in the presence of Dox. The cells were stained with DAPI (blue) and anti-P-AMPK (red) antibody. The mitotic cells at metaphase were observed. The signal of expressed PRL (WT or C104S) was detected via the N-terminally tagged GFP. Scale bar: 5 μm. (C) Control MDCK cells or Dox-inducible PRL3-expressing MDCK cells cultured under pH-fixed condition (pH 7.5) for 14 h in the presence of Dox were treated either with A769662 (300 μM) or AICAR (5 mM) for 30 min before fixation. Scale bar: 5 μm. (D) Quantification of the spindle angle. Data are shown as mean ± SEM, n = 50 cells. The *p* values were calculated via one-way ANOVA with Tukey’s multiple comparison. ****p<0.0001 against cells without inhibitor treatment. (E) MDCK cells cultured under pH-fixed condition (pH 7.5) for 14 h were treated with compound C (20 μM) for the indicated time. The cells were stained with DAPI (blue) and anti-P-AMPK (red) antibody. Scale bar: 5 μm. (F) Quantification of the spindle angle. Data are shown as mean ± SEM, n = 30 cells. The *p* values were calculated via one-way ANOVA with Tukey’s multiple comparison. **p<0.01, ****p<0.0001 against control cells.

We previously reported that PRL suppresses the AMPK activation through the upregulation of intracellular Mg^2+^ and ATP levels (Funato *et al.*, 2014). Moreover, multiple studies have shown that spindle misorientation and mitotic delay can be caused by AMPK dysfunction (Thaiparambil *et al.*, 2012; Lee *et al.*, 2007; Stauffer *et al.*, 2019). This led us to examine the levels of P-AMPK, an activated form of AMPK, in mitotic cells. The cells were treated with nocodazole to synchronize them during mitosis, and then the levels of P-AMPK were determined by western blotting analysis (Supplementary Fig. 3). P-AMPK expression levels were increased during mitosis, similar to cyclin B1 levels, but no significant difference was observed between control and PRL3-expressing cells. Therefore, we performed immunofluorescence staining for P-AMPK to investigate its localization in mitotic cells. During mitosis, P-AMPK is known to localize to several mitotic structures, such as centrosomes, and spindle poles, midzones, and midbodies, through prophase to cytokinesis (Vazquez-Martin *et al.*, 2011; Lu *et al.*, 2021). Our observations confirmed P-AMPK localization, but additional dot-like signals of P-AMPK at the chromosomes in mitotic cells from prometaphase to anaphase were also observed (Fig. 4A). These signals were considered to be localized at the kinetochores because they overlapped with the CREST signals. Such kinetochore-localized P-AMPK signals were also observed in HeLa cells, although the signals were mostly observed during prometaphase (Supplementary Fig. 4). Moreover, a similar localization of P-AMPK at the kinetochore has been reported in mouse intestinal epithelial cells and culture cell lines such as HeLa and U2OS (Lu *et al.*, 2021; Wei *et al.*, 2012). To investigate whether PRL3 expression affects the localization of P-AMPK signals, cells were stained for P-AMPK (Fig 4B). The results showed that kinetochore-localized P-AMPK signals were reduced in PRL3-expressing cells compared to control, and PRL3-C104S expressing cells, but no significant differences were observed in other mitotic structures such as spindle poles. In addition, kinetochore-localized P-AMPK signals in PRL3-expressing cells were restored by placing cells under acidic condition of pH 6.5 (Fig. 4B). Next, we treated PRL3-expressing cells with two different AMPK activators, A769662 and AICAR, and found that they restored P-AMPK signals at the kinetochores and spindle orientation to the level of control cells (Fig. 4C–D). We also tested the effect of compound C, an AMPK inhibitor, on control cells (Fig. 4E). When it was used at 20 μM for 10–20 min, P-AMPK signals at the spindle poles were still maintained, but kinetochore-localized signals almost disappeared. Under these conditions, the spindle angle was increased (Fig. 4F). Collectively, these data indicate that PRL3-expression reduces P-AMPK levels, specifically at kinetochores, and suggest its role in mitotic fidelity.

### PRL3 expression disturbs recruitment of SAC proteins at kinetochores

**Fig. 5:**
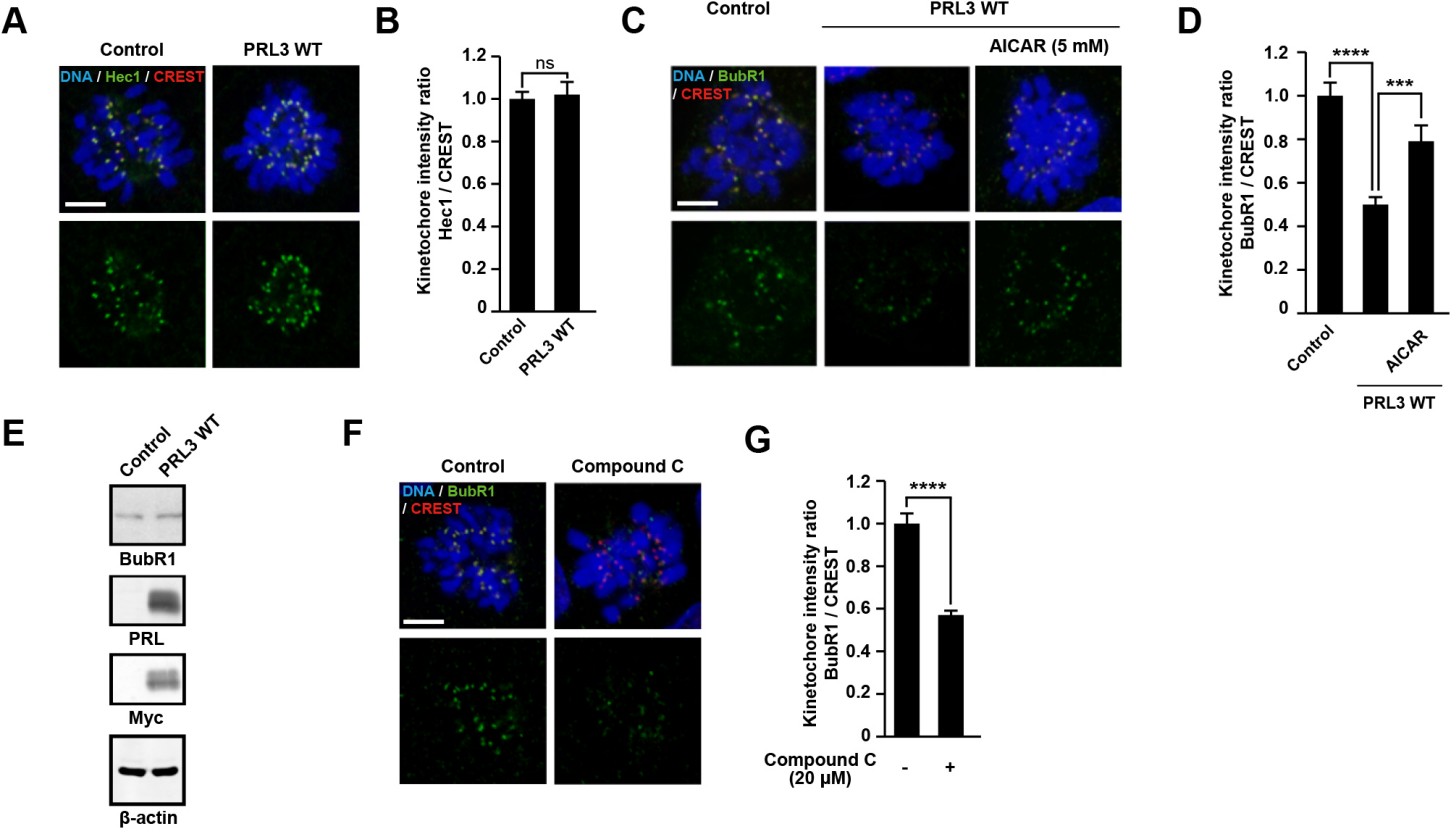
PRL3 perturbs kinetochore recruitment of BubR1 (A) Control MDCK cells or Dox-inducible PRL3-expressing MDCK cells cultured under pH-fixed condition (pH 7.5) in the presence of Dox. The cells were stained with DAPI (blue), anti-Hec1 (green), and anti-CREST (red) antibodies. The mitotic cells at prometaphase were observed. Scale bar: 5 μm. (B) Quantification of Hec1 levels relative to CREST levels at kinetochores. Data are shown as mean ± SEM, n = 30 cells. The *p* values were calculated via two-tailed unpaired *t*-test. ns: non-significant against control cells. (C) Control MDCK cells or Dox-inducible PRL3-expressing MDCK cells were cultured under pH-fixed condition (pH 7.5) in the presence of Dox were treated or untreated with 5 mM AICAR for 30 min before fixation. Cells were stained with DAPI (blue), anti-BubR1 (green), and anti-CREST (red) antibodies. Scale bar: 5 μm. (D) Quantification of BubR1 levels relative to CREST levels at kinetochores. Data are shown as mean ± SEM, n = 30 cells. The *p* values were calculated via one-way ANOVA with Tukey’s multiple comparison. ***p<0.001, ****p<0.0001. (E) Cell lysates were subjected to SDS-PAGE and immunoblotting with the indicated antibodies. (F) MDCK cells cultured under pH-fixed condition (pH 7.5) for 14 h were treated with compound C (20 μM) for 20 min before fixation. The cells were stained with DAPI (blue), anti-BubR1 (green), and anti-CREST (red) antibodies. Scale bar: 5 μm. (G) Quantification of BubR1 levels relative to CREST levels at kinetochores. Data are shown as mean ± SEM, n = 30 cells. The *p* values were calculated via two-tailed unpaired *t*-test. ****p<0.0001.

The kinetochore is a large protein complex that links chromosomes to spindle microtubules emanating from the spindle poles (Cheeseman and Desai, 2008). During prometaphase, constitutive centromere-associated network (CCAN) proteins recruit Knl1, Mis12, and Ndc80/Hec1 (KMN) network proteins, which act as platforms for spindle assembly checkpoint (SAC) proteins that prevent mitosis progression until the microtubules and chromosomes attach correctly (Foley and Kapoor, 2013). We first stained cells for Hec1, an essential component of the KMN network proteins, during prometaphase (Fig. 5A) and found no significant difference between control and PRL3-expressing cells (Fig. 5B). We next investigated the localization of BubR1, a SAC protein, which showed a reduction in its signal intensities at kinetochores in PRL3-expressing cells (Fig. 5C–D), without significant differences in the total protein levels (Fig. 5E). Consistent with the results for BubR1, immunofluorescence and immunoblotting analyses of Mad2, another SAC protein, also showed similar results (Supplementary Fig. 5A–C). Collectively, we concluded that PRL3 expression perturbs the recruitment of SAC proteins to kinetochores, which affects chromosome assembly. Next, to investigate the importance of AMPK in recruiting SAC proteins to kinetochores, cells were treated with compound C and then stained for SAC proteins, BubR1 and Mad2, to observe their signals at kinetochores (Fig. 5F, Supplementary Fig. 5D). The results showed that the intensities of SAC protein signals at kinetochores were reduced following treatment with compound C (Fig. 5G, Supplementary Fig. 5E). Furthermore, kinetochore-localized BubR1 and Mad2 were restored after treatment of an activator of AMPK, AICAR, in PRL3-expressing cells (Fig. 5C–D, and Supplementary Fig. 5A–B). Taken together, these data indicate that PRL3 expression perturbs the recruitment of SAC proteins to kinetochores by suppressing the activation of kinetochore-localized AMPK, resulting in inappropriate chromosomal alignment.

## Discussion

In this study, we showed that PRL overexpression specifically inhibits the recruitment of P-AMPK at kinetochore during mitosis without affecting its localization to other mitotic structures. Although several studies have reported that P-AMPK localizes at the kinetochore (Lu *et al.*, 2021; Wei *et al.*, 2012), its functional importance and localization mechanism remain largely unknown. Our observations demonstrated the importance of PRL in this process and revealed the events of mitotic errors associated with AMPK function.

We found that PRL overexpression perturbed the recruitment of SAC proteins (BubR1 and Mad2) at the kinetochore, while KMN network protein (Hec1) was normally recruited. Therefore, we believe that AMPK phosphorylation of some kinetochore proteins is important for the recruitment of SAC proteins. A previously reported phosphoproteomic analysis showed 131 phosphorylation sites for AMPK, and the phosphorylation level of 31 of these sites were increased specifically in mitosis (Schaffer *et al.*, 2015). Among these, only APC has been shown to localize at the kinetochore during mitosis (Fodde *et al.*, 2001; Kaplan *et al.*, 2001). APC plays a role in mitosis and regulates the interaction between kinetochores and microtubules (Fodde *et al.*, 2001; Kaplan *et al.*, 2001), which is essential for proper mitotic progression. Consistently, APC depletion by RNAi perturbed the recruitment of SAC proteins at the kinetochore and enhanced lagging chromosome and aneuploidy (Draviam *et al.*, 2006). Therefore, APC is considered one of the candidate proteins that might be phosphorylated by AMPK and involved in the recruitment of SAC proteins at kinetochores during mitosis. Clarification of the molecular details downstream of P-AMPK at kinetochores is important for future studies.

An important question that remains unanswered is how PRL affects AMPK function. It was previously reported that PRL1 knockdown by RNAi upregulates AMPK phosphorylation levels by immunoblotting of cell lysates (Funato *et al.*, 2014), which led us to hypothesize that PRL expression suppresses AMPK phosphorylation. However, immunoblotting analysis did not reveal a significant decrease in P-AMPK levels (Supplementary Fig. 3). Several studies have reported that P-AMPK levels increase during mitosis (Vazquez-Martin *et al.*, 2011; Lu *et al.*, 2021; Thaiparambil *et al.*, 2012). Therefore, we also performed a P-AMPK immunoblotting analysis using lysates of cells synchronized at mitosis; however, no significant difference was observed (Supplementary Fig. 3). Since P-AMPK is known to localize to various mitotic structures, we performed immunofluorescence analyses of cells undergoing mitosis. Interestingly, P-AMPK-staining signals specifically decreased at the kinetochore in PRL-expressing cells (Fig. 4B). Furthermore, treatment of PRL-expressing cells with AMPK activators, A769662 and AICAR, restored not only P-AMPK signals at kinetochore but also spindle orientation to levels in control cells (Fig. 4C). These results collectively imply that PRL regulates the levels of P-AMPK specifically at the kinetochore, and kinetochore-localized P-AMPK plays an important role in mitotic fidelity. It remains unknown how PRL expression alters P-AMPK levels at the kinetochore, which is an important topic for future studies.

PRL has a tyrosine phosphatase domain and a weak but significant intrinsic phosphatase activity (Kozlov *et al.*, 2004). PRL has been reported to directly interact with CNNM Mg^2+^ transporters and inhibit Mg^2+^ efflux activity (Funato *et al.*, 2014; Hardy *et al.*, 2015; Gulerez *et al.*, 2016). We performed analyses using colonic epithelial cells from *Cnnm4*-KO mice, including the tissue sections and crypt-derived organoids, and found that they had higher levels of mitotic errors (Fig. 3), similar to PRL-expressing MDCK cells. Therefore, among the two PRL protein activities, the CNNM binding ability seems to be important in inducing mitotic errors. It should also be noted that this conclusion is consistent with previous studies showing that the interaction between PRL and CNNM Mg^2+^ transporters is essential for PRL-induced cancer malignancy (Funato *et al.*, 2014; Hardy *et al.*, 2015; Gulerez *et al.*, 2016; Kozlov *et al.*, 2020; Hardie, 2007). Clarification of the molecular details of the dysfunction in Mg^2+^ regulation by CNNM proteins and the AMPK activation at the kinetochore during mitosis is critical for understanding the role of PRL in progression of cancer malignancy.

## Abbreviations

AMPK: AMP-activated protein kinase
BSA: bovine serum albumin
CAMKK: calcium/calmodulin-dependent protein kinase kinase
CCAN: constitutive centromere-associated network
CNNM: cyclin M
DAPI: 6-diamidino-2-phenylindole
DMEM: Dulbecco’s modiﬁed Eagle’s medium
Dox: doxycycline
F-actin: filamentous actin
FBS: fetal bovine serum
H&E: hematoxylin and eosin
KMN: network Knl1 Mis12 and Ndc80/Hec1 network
KO: knockout
LKB1: liver kinase B1
Noco: nocodazole
P-AMPK: phosphorylated AMPK
PFA: paraformaldehyde
PRL: phosphatase of regenerating liver
RT: room temperature
SAC: spindle assembly checkpoint
TX-100: TritonX-100
WT: wild-type
