# Supplementary material for: PRL stimulates mitotic errors by suppressing kinetochore-localized activation of AMPK during mitosis
Source: Cell Struct Funct. 2022 Nov 5;47(2):75–87. doi: 10.1247/csf.22034 (PMC10511051; doi:10.1247/csf.22034)
Supplement: Supplementary file 1 — Supplementary Fig. 1 [file csf_47_22034_1.pdf]

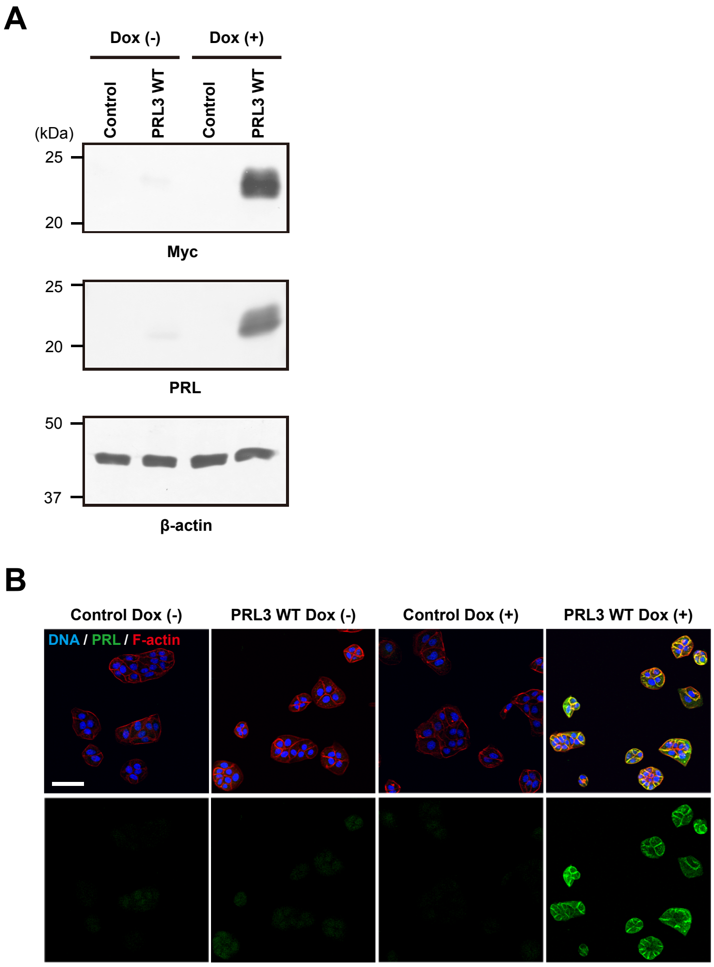

Supplementary Figure 1. Establishment of Dox-inducible Myc-PRL3-expressing cells

(A) Control MDCK cells or Dox-inducible PRL3-expressing MDCK cells were cultured under pH-fixed condition (pH 7.5) for 14 h in the presence (+) or absence (-) of Dox. Cell lysates were subjected to SDS-PAGE and immunoblotting with the indicated antibodies. (B) The cells were stained with DAPI (blue), anti-Myc antibody (green), and rhodamine-conjugated phalloidin (red). Lower panels show the fluorescence signal of Myc-PRL3. Scale bar: 50 µm.
