# Supplementary material for: PRL stimulates mitotic errors by suppressing kinetochore-localized activation of AMPK during mitosis
Source: Cell Struct Funct. 2022 Nov 5;47(2):75–87. doi: 10.1247/csf.22034 (PMC10511051; doi:10.1247/csf.22034)
Supplement: Supplementary file 2 — Supplementary Fig. 2 [file csf_47_22034_2.pdf]

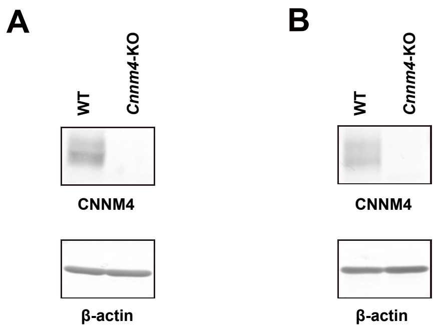

Supplementary Figure 2. Verification of *Cnnm4*-KO by immunoblotting analysis

Lysates of the colon tissues (A) and the colon-derived organoids (B) were subjected to SDS-PAGE and immunoblotting with the indicated antibodies.
