# Supplementary material for: PRL stimulates mitotic errors by suppressing kinetochore-localized activation of AMPK during mitosis
Source: Cell Struct Funct. 2022 Nov 5;47(2):75–87. doi: 10.1247/csf.22034 (PMC10511051; doi:10.1247/csf.22034)
Supplement: Supplementary file 3 — Supplementary Fig. 3 [file csf_47_22034_3.pdf]

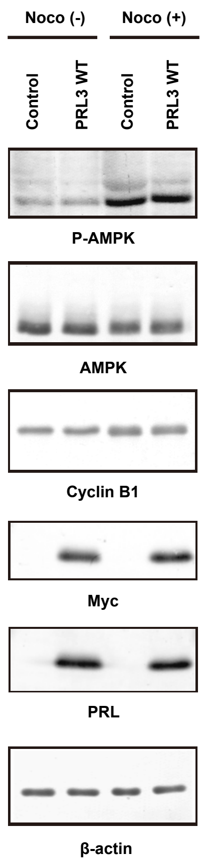

Supplementary Figure 3. No significant changes in P-AMPK levels by PRL3 expression

Control MDCK cells or Dox-inducible PRL3-expressing MDCK cells cultured under pH-fixed condition (pH 7.5) in the presence of Dox were treated (+) or untreated (-) with 500 nM nocodazole (noco) for 16 h. Cell lysates were subjected to SDS-PAGE and immunoblotting with the indicated antibodies.
