# Supplementary material for: PRL stimulates mitotic errors by suppressing kinetochore-localized activation of AMPK during mitosis
Source: Cell Struct Funct. 2022 Nov 5;47(2):75–87. doi: 10.1247/csf.22034 (PMC10511051; doi:10.1247/csf.22034)
Supplement: Supplementary file 4 — Supplementary Fig. 4 [file csf_47_22034_4.pdf]

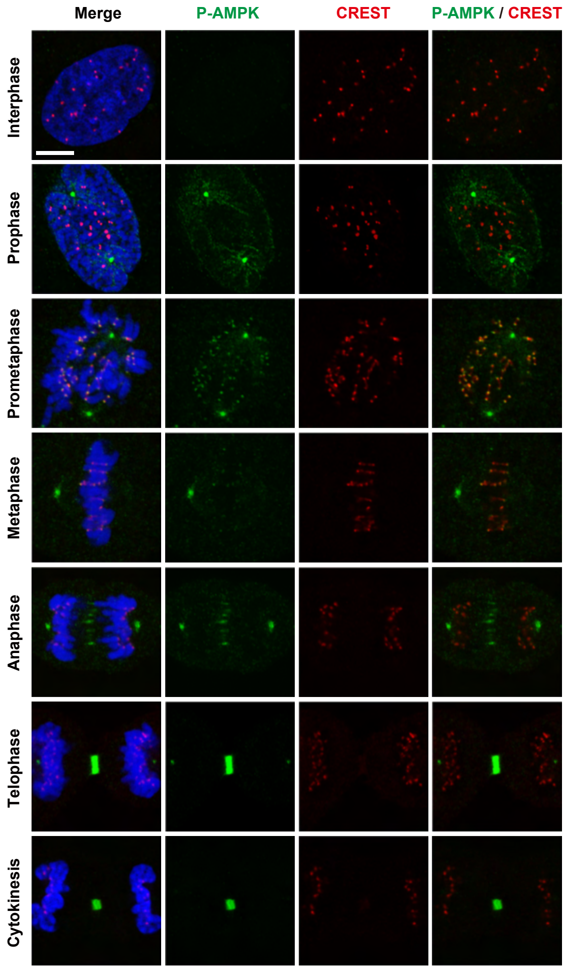

Supplementary Figure 4. P-AMPK localization in HeLa cells

HeLa cells were cultured under pH-fixed condition (pH 7.5) for 14 h. Cells were stained with DAPI (blue), anti-P-AMPK (green), and anti-CREST (red) antibodies. The cells at indicated phases were observed. Scale bar: 5 μm.
