# Supplementary material for: PRL stimulates mitotic errors by suppressing kinetochore-localized activation of AMPK during mitosis
Source: Cell Struct Funct. 2022 Nov 5;47(2):75–87. doi: 10.1247/csf.22034 (PMC10511051; doi:10.1247/csf.22034)
Supplement: Supplementary file 5 — Supplementary Fig. 5 [file csf_47_22034_5.pdf]

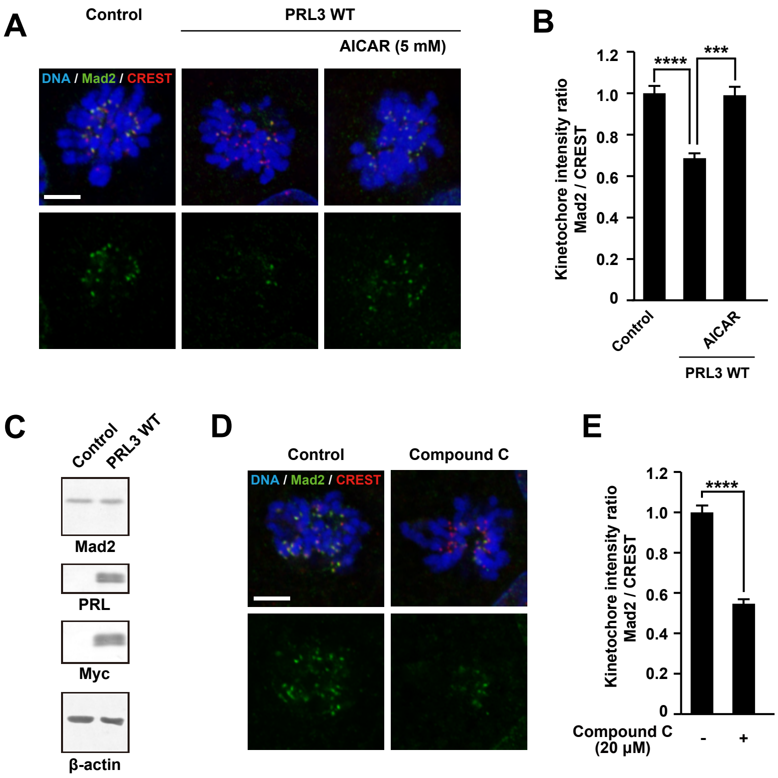

Supplementary Figure 5. PRL3 perturbs kinetochore recruitment of Mad2

(A) Control MDCK cells or Dox-inducible PRL3-expressing MDCK cells were cultured under pH-fixed condition (pH 7.5) for 14 h in the presence of Dox were treated or untreated with 5 mM AICAR for 30 min before fixation. The cells were stained with DAPI (blue), anti-Mad2 (green), and anti-CREST (red) antibodies then mitotic cells at prometaphase were observed. Lower panels show fluorescence signal of Mad2. Scale bar: 5 μm. (B) Quantification of Mad2 levels relative to CREST levels at kinetochores. Data are shown as mean ± SEM, n = 30 cells. The p values were calculated via one-way ANOVA with Tukey's multiple comparison. ***$p < 0.001$, ****$p < 0.0001$ against control cells. (C) Control MDCK cells or Dox-inducible PRL3-expressing MDCK cells were cultured under pH-fixed condition (pH 7.5) for 14 h in the presence of Dox. Cell lysates were subjected to SDS-PAGE and immunoblotting with the indicated antibodies. (D) MDCK cells cultured under pH-fixed condition (pH 7.5) for 14 h were treated with compound C (20 μM) for 20 min before fixation. The cells were stained with DAPI (blue), anti-Mad2 (green), and anti-CREST (red) antibodies. Lower panels show fluorescence signal of Mad2. (E) Quantification of Mad2 levels relative to CREST levels at kinetochores. Data are shown as mean ± SEM, n = 30 cells. The p values were calculated via two-tailed unpaired t-test. ****$p < 0.0001$ against control cells.
