# Supplementary material for: PRL stimulates mitotic errors by suppressing kinetochore-localized activation of AMPK during mitosis
Source: Cell Struct Funct. 2022 Nov 5;47(2):75–87. doi: 10.1247/csf.22034 (PMC10511051; doi:10.1247/csf.22034)
Supplement: Supplementary file 6 — Supplementary Table 1 [file csf_47_22034_6.zip › Supplementary_Table_1.docx]

**Supplementary Table 1. MITOTIC CATASTROPHE AND DURATION OF MITOSIS FROM TIME-LAPSE EXPERIMENT**

|  | Control | | PRL | |
| --- | --- | --- | --- | --- |
|  | Mitotic catastrophe (O/X) | Duration of mitosis (min) | Mitotic catastrophe (O/X) | Duration of mitosis (min) |
|  | X | 20 | X | 100 |
|  | X | 20 | O | ND |
|  | X | 20 | O | ND |
|  | X | 20 | O | ND |
|  | X | 40 | X | 80 |
|  | X | 40 | O | ND |
|  | X | 60 | X | 100 |
|  | X | 40 | X | 100 |
|  | X | 120 | O | ND |
|  | X | 40 | O | ND |
|  | X | 80 | O | ND |
|  | X | 40 | X | 80 |
|  | X | 60 | O | ND |
|  | X | 100 | O | ND |
|  | X | 80 | O | ND |
|  | X | 100 | O | ND |
|  | X | 60 | O | ND |
|  | X | 80 | O | ND |
|  | X | 20 | X | 80 |
|  | X | 80 | O | ND |
| Total | 0/20 | NA | 14/20 | NA |
| Average (min) | NA | 56 | NA | 90^***^ |

Note: Frequency of mitotic catastrophe (%) and duration of mitosis (min) from DNA condensation until the cell die or divide in 20 mitotic cells in control MDCK cells or Dox-inducible PRL3-expressing cells. Data are shown as mean ± SEM, n = 20 mitotic cells. The *p* value was calculated via two-tailed unpaired *t*-test with Welch’s correction. NA = not available, ND = not determined, ****p* < 0.001 against control cells.
